# Effect of conductive hearing loss on central auditory function^☆^

**DOI:** 10.1016/j.bjorl.2016.02.010

**Published:** 2016-04-22

**Authors:** Arash Bayat, Mohammad Farhadi, Hesam Emamdjomeh, Nader Saki, Golshan Mirmomeni, Fakher Rahim

**Affiliations:** a Ahvaz Jundishapur University of Medical Sciences, Hearing and Speech Research Center, Ahvaz, Iran; b Iran University of Medical Sciences, Hazrat Rasoul Akram Hospital, Head and Neck Surgery, Department and Research Center of Otolaryngology, Tehran, IR, Iran; c Iran University of Medical Sciences, Haztat Rasoul Akram Hospital, Department of Audiology, Tehran, IR, Iran; d Ahvaz Jundishapur University of Medical Sciences, Student Research Committee, Ahvaz, Iran

**Keywords:** Adult, Auditory temporal processing, Conductive hearing loss, Gap in noise, Adulto, Processamento temporal auditivo, Perda auditiva condutiva, *Gap* no ruído

## Abstract

**Introduction:**

It has been demonstrated that long-term Conductive Hearing Loss (CHL) may influence the precise detection of the temporal features of acoustic signals or Auditory Temporal Processing (ATP). It can be argued that ATP may be the underlying component of many central auditory processing capabilities such as speech comprehension or sound localization. Little is known about the consequences of CHL on temporal aspects of central auditory processing.

**Objective:**

This study was designed to assess auditory temporal processing ability in individuals with chronic CHL.

**Methods:**

During this analytical cross-sectional study, 52 patients with mild to moderate chronic CHL and 52 normal-hearing listeners (control), aged between 18 and 45 year-old, were recruited. In order to evaluate auditory temporal processing, the Gaps-in-Noise (GIN) test was used. The results obtained for each ear were analyzed based on the gap perception threshold and the percentage of correct responses.

**Results:**

The average of GIN thresholds was significantly smaller for the control group than for the CHL group for both ears (right: *p* = 0.004; left: *p* < 0.001). Individuals with CHL had significantly lower correct responses than individuals with normal hearing for both sides (*p* < 0.001). No correlation was found between GIN performance and degree of hearing loss in either group (*p* > 0.05).

**Conclusion:**

The results suggest reduced auditory temporal processing ability in adults with CHL compared to normal hearing subjects. Therefore, developing a clinical protocol to evaluate auditory temporal processing in this population is recommended.

## Resumo

**Introdução:**

Já foi demonstrado que a perda auditiva condutiva (PAC), em longo prazo, pode influenciar na detecção precisa das características temporais dos sinais acústicos ou do processamento auditivo temporal (PAT). Pode-se argumentar que o PAT pode ser o componente subjacente de muitos recursos do processamento auditivo central, como a compreensão da fala ou localização do som. Pouco se sabe sobre as consequências da PAC nos aspectos temporais do processamento auditivo central.

**Objetivo:**

Este estudo foi projetado para avaliar a capacidade de processamento auditivo temporal em indivíduos com PAC crônica.

**Método:**

Durante este estudo transversal analítico, 52 pacientes com PAC crônica leve a moderada e 52 indivíduos com audição normal (controle), idades entre 18 e 45 anos, foram recrutados. Para avaliar o processamento auditivo temporal, foi utilizado o teste de resolução temporal *Gaps-in-Noise* (GIN). Os resultados obtidos para cada orelha foram analisados com base no limiar de percepção da quebra de continuidade (*gap*) e na porcentagem de respostas corretas.

**Resultados:**

A média dos limiares no GIN foi significativamente menor para o grupo controle que para o grupo PAC em ambas as orelhas (direita: *p* = 0,004; esquerda: *p* < 0,001). Os indivíduos com PAC apresentaram respostas corretas significativamente mais baixas que os indivíduos com audição normal em ambas as orelhas (*p* < 0,001). Não houve correlação entre o desempenho no GIN e o grau de perda auditiva em ambos os grupos (*p* > 0,05).

**Conclusão:**

Os resultados sugerem uma redução da capacidade de processamento auditivo temporal em adultos com PAC comparados com indivíduos apresentando audição normal. Portanto, o desenvolvimento de um protocolo clínico para avaliar o processamento auditivo temporal nessa população é recomendado.

## Introduction

Chronic Conductive Hearing Loss (CHL) is characterized by reduced efficiency of sound transmission through the external and/or middle ear and usually involves a reduction in sound level or the ability to hear faint sounds. Several investigators have argued that this long-term sensory deprivation may produce irreversible changes in the anatomical and functional integrity of the central auditory structures,^1^, ^2^, ^3^ such as changes in the relative size of neuron dendrites in subcortical nuclei^4^, ^5^ or synaptic and spike adaptation disruptions in the auditory cortex.^6^

It has been also demonstrated that auditory deprivation following CHL may be associated with a number of sensory and cognitive difficulties as well as deficits in psychosocial development.^6^, ^7^, ^8^ These problems may continue long after hearing thresholds return to normal limits.

CHL may influence the accurate processing of the time structure of the acoustic signal, e.g. delays low frequency sounds entering the inner ear by up to 150 μs.^9^ Auditory Temporal Processing (ATP), one of the (central) auditory processing mechanisms, refers to the ability of the auditory system to process temporal characteristics of a sound stimulus within a specific time period.^2^, ^5^, ^10^, ^11^ It can be argued that ATP may be the underlying component of many auditory processing capabilities, including the processing of speech transients and voicing information, segregation of auditory figure from auditory ground and localization cues,^12^, ^13^ and being a prerequisite for speech and language acquisition.^14^ This notion can be observed at different levels ranging from the neuronal sensitivity of first order neurons to the cortical level.^15^, ^16^

The Gaps-In-Noise (GIN) test provides a clinically feasible method of assessing ATP, temporal resolution, wherein the subjects are required to detect gaps within a continuous auditory stimulus.^3^, ^17^ This test could be easily administered and performed using common equipment, and used for a wide age range (beginning from 7 years of age). The GIN yields good sensitivity (74%) and specificity (94%) to central auditory nervous system dysfunction in adult populations while still demonstrating clinical feasibility.^15^ It has been shown that GIN is more sensitive to cortical compromise as opposed to brainstem deficits.^15^

Aravindkumar et al. ^7^ reported bilaterally impaired temporal processing ability in their study of 26 patients with refractory complex partial seizures and Mesial Temporal Sclerosis (MTS). Patients were divided into two groups: right MTS (*n* = 13; mean age: 31 years) and left MTS (*n* = 13; mean age: 25.76 years). Fifty healthy subjects (mean age: 26.3 years) constituted the control group. They reported that both MTS groups showed longer GIN thresholds and less percentage of correct responses in both ears when compared to the control group. These findings show that GIN is sensitive to cortical lesions.

While it has been known that a CHL can distort auditory processing, the effect of CHL on temporal aspects of auditory processing has received little attention. The purpose of this study was to investigate the effects of CHL on auditory temporal ability by employing the GIN test.

## Methods

### Participants

This was an analytical cross-sectional study approved by the Local Research Ethics Committee, and informed consent was obtained from all participants. The sample consisted of 104 adults aged 18–45 years (mean: 27.02 years) who were referred to the otolaryngology department in a general hospital. They were selected in a consecutive sampling method and classified into two groups:

**Control group** (*n* = 52): This group was composed of healthy subjects without a history of otitis media and showed a threshold of 10 dB HL or less for octave frequencies between 250 Hz and 8000 Hz, bilaterally. Tympanograms were recorded normally (Type An) in both ears.

**CHL group** (*n* = 52): This group had bilateral symmetrical mild to moderate CHL with pure-tone averages (500, 1000, 2000 Hz) ranging from 34 to 51 dB HL and monosyllabic word recognition scores in quiet of 90% or greater. The onset of disease was greater than two weeks and the chief complaint was hearing loss sensation. The patients were evaluated using otomicroscopic and Computed Tomography (CT) examinations, and subjects with cholesteatoma or craniofacial abnormalities were excluded.

Both groups were matched for age and gender. All subjects were right-handed and native speakers of Persian and showed normal scores on the Mini-Mental State Examination (MMSE). The MMSE is a useful screening instrument for assessing global cognitive function. It evaluates five areas of cognitive function: orientation, registration, attention and calculation, recall, and language. Scoring on this test varies from 0 to 30 points; higher scores indicate better cognitive functioning. A cut-off of 23 points was used for our Iranian sample.^16^

Individuals with a history of metabolic, psychiatric, developmental or neurological problems were also excluded from the study.

### Procedures

All participants were tested while seated in a double-walled sound-treated booth. Pure-tone audiometry was performed using a calibrated audiometer (Amplaid A321, Italy), and frequencies from 250 to 8000 Hz were tested using the ascending–descending method with a step size of 5 dBHL.

The GIN stimuli, which were recorded on a compact disc, were played on a Sony DVPN728H DVD player and passed through the speech circuitry of an audiometer to TDH-39 matched headphones. The stimuli were presented monaurally at 45 dB SL (relative to the mean pure tone thresholds at 500, 1000 and 2000 Hz) and the test duration was approximately 16 min for each participant. The GIN lists 1, 2 and 3 were applied, alternately, in the right and left ears of each individual. Subjects were asked to identify the gaps distributed throughout 6 seconds of white noise presentation. Each test list was composed of 0–3 silent intervals or gaps contained within each 6-second segment of white noise. The interstimulus interval between noise segments was 5 s. The duration of each gap was 2, 3, 4, 5, 6, 8, 10, 12, 15 or 20 ms, and they were randomly distributed so that 60 gaps (6 of each duration) were presented in each list. Eight practice items precede the administration of the test items.^15^ The participants were instructed to press the response button as soon as they perceived a gap or a silence in the noise segments presented.

The results obtained for each ear were analyzed based on the gap perception threshold and the percentage of correct responses. The GIN threshold was defined as the shortest gap duration for which there were at least 4 of 6 correct identifications. Since there were 60 gaps in each list, the percentage of correct responses was defined and calculated as the percentage of correct responses scored across all gaps^15^: (Total number of gaps identified/total number of gaps in the list) × 100.

The Statistical Package for the Social Sciences (SPSS) for Windows was used for statistical analysis. The inferential statistical analyses were conducted using parametric tests, since, after submitting the data set to the Kolmogorov–Smirnov Test (for normal distribution of data) and the Levene's Test (for homogeneity of variances), we found that the results met the requisites for the application of parametric tests. Therefore, the independent sample *t*-test and paired *t*-test were used. The level of statistical significance was set at 0.05.

## Results

In the CHL group, the mean Pure Tone Average (PTA) threshold for the right and left ears were 41.03 ± 7.29 dBHL and 40.89 ± 8.37 dBHL, respectively. In the control group, the mean PTA threshold for the right and left sides were 6.08 ± 3.44 dBHL and 5.56 ± 4.15 dBHL, respectively.

The GIN test results were analyzed according to the percentage of correct responses and to the gap detection threshold. Table 1 shows the mean percentage of correct identification scores for both groups. A review of these data showed higher (better) percentages of correct responses for both the left and right ears for control group when compared with the performance of the CHL group for the total number of percent correct responses to the GIN test. The comparison of GIN scores between the ears showed no significant differences between ears in either group (control group, *p* = 0.33; CHL group, *p* = 0.19).

**Table 1:** Comparison of the percentage of correct responses (%) between the control and Conductive Hearing Loss (CHL) groups.

Ear	Control group		CHL group		*p*-Value
	Mean	SD	Mean	SD	
Right	72.88	6.89	63.95	9.51	<0.001
Left	73.54	6.12	64.30	8.24	<0.001

Table 2 shows the comparison of the GIN threshold between the control and the CHL groups. The results of the independent sample *t*-test revealed that the mean GIN thresholds were significantly smaller (better) for the control group than for the CHL group for both ears. The results of the paired *t*-test showed no significant differences between the ears in either group, which indicates similarity of responses between ears in each group (control group, *p* = 0.51; CHL group, *p* = 0.21).

**Table 2:** Comparison of the GIN threshold (ms) between the control and Conductive Hearing Loss (CHL) groups.

Ear	Control group		CHL group		*p*-Value
	Mean	SD	Mean	SD	
Right	4.29	0.53	6.53	1.70	0.004
Left	4.21	0.66	6.95	1.82	<0.001

### Inter-list comparisons

Two lists of 60 GIN items per list were used in the current study. Thirty control subjects were administered three lists (list 1, list 2 and list 3) in random order to establish inter-list equivalence. A one-way analysis of variance demonstrated no significant differences across lists for either ear (*p* = 0.12).

## Discussion

The results of the current study indicated that individuals with CHL need a longer duration to detect gaps on the GIN test compared to individuals with normal hearing sensitivity. Additionally, we found a higher percentage of correct responses in control listeners versus CHL subjects. The finding of reduced gap detection ability in CHL patients agrees with findings of Balen et al. ^18^ These authors compared the temporal resolution ability of children with normal hearing (*n* = 12), with those bearing CHL (*n* = 7) and auditory processing disorders (*n* = 12) using Random Gap Detection Test (RGDT). Their findings demonstrated that children with hearing impairment exhibited significantly higher gap detection thresholds than those children with normal hearing sensitivity. However, they stated that RGDT test has great performance variability in assessing the auditory temporal resolution.

The ability to make fine temporal discriminations of acoustic signals is an important element in analyzing the characteristics of sensory input and contributes to a number of auditory perceptions, including certain speech comprehension situations and sound localization.^19^, ^20^ Neurons along the central auditory pathways maintain the precise timing of spikes, which is attributable to specialized synaptic mechanisms (for example, via calyx synapses, the largest synapses in the auditory brainstem) and biophysical membrane properties.^21^, ^22^, ^23^ It seems that these characteristics are very important for detecting the acoustic features that change on millisecond time scales.^6^

It has been shown that the central part of the auditory system responds dynamically to the level of neural input it receives from the ears. Xu et al. ^6^ revealed that CHL significantly alters temporally-precise properties of auditory cortex synapses and spikes, and this may contribute to ATP deficits caused by mild to moderate hearing loss. These findings show that the auditory system responds dynamically to the level of neural input it receives from the ears.

Musiek et al. ^15^ showed that the GIN test is sensitive in confirming lesions of the central auditory nervous system, being even more sensitive to cortical damage. In the present study, the difference found between the two groups in the performance on the GIN test indicates central auditory nervous system dysfunction in patients with chronic CHL.

In the present study, signals were presented at an equal sensation level for all listeners, which is signal level re-absolute threshold. Stimulus level is known to be an important factor in psychophysical assessments such as temporal resolution tests. In some occasions, subject's performance may improve with an increase in stimulus level until asymptotic performance is achieved.^18^, ^24^

The GIN test results in our study showed a similar performance in test lists 1, 2 and 3 regardless of which ear started the exam. These findings indicate no learning effect or fatigue, as has been observed in other similar studies.^11^

No advantage of one ear over the other in GIN thresholds and percentage of correct answers were observed. Our results are in agreement with other published papers where no perceptual asymmetry between the ears was reported for gap detection.^11^, ^15^ However, Sininger and de Bode^25^ found a smaller left-ear advantage for gap detection using tonal stimuli.

## Conclusion

The findings of the current investigation showed that auditory temporal resolution ability has been impaired in individuals with CHL versus normal hearing subjects. Therefore, developing a clinical protocol to evaluate auditory temporal processing in this population is highly recommended. Furthermore, identification of such central auditory disorders in patients with hearing loss would provide a better insight to more effective interventions.

## Conflicts of interest

The authors declare no conflicts of interest.
